# Selective Fluorescent Probes for High-Throughput Functional Diagnostics of the Human Multidrug Transporter P-Glycoprotein (ABCB1)

**DOI:** 10.3390/ijms231810599

**Published:** 2022-09-13

**Authors:** Edit Szabó, Anna Kulin, Bálint Jezsó, Nóra Kucsma, Balázs Sarkadi, György Várady

**Affiliations:** 1 Institute of Enzymology, Research Centre for Natural Sciences, 1117 Budapest, Hungary; 2 Doctoral School of Molecular Medicine, Semmelweis University, 1085 Budapest, Hungary; 3 Department of Biophysics and Radiation Biology, Semmelweis University, 1094 Budapest, Hungary

**Keywords:** ABC transporter, ABCB1, P-glycoprotein, functional biology, multidrug transporter, TO-PRO-3, TO-PRO-1, cyanine dye

## Abstract

The multidrug transporter ABCB1 (MDR1, Pgp) plays an important role in the absorption, distribution, metabolism, and elimination of a wide range of pharmaceutical compounds. Functional investigation of the ABCB1 expression is also essential in many diseases, including drug-resistant cancer, inflammatory conditions, or Alzheimer disease. In this study, we examined the potential interaction of the ABCB1 multidrug transporter with a group of commercially available viability dyes that are generally considered not to penetrate into intact cells. Here, we demonstrate that the slow cellular accumulation of TO-PRO™-1 (TP1) or TO-PRO™-3 (TP3) was strongly inhibited by ABCB1-dependent dye extrusion. TP1/3 dye accumulation was not affected by the presence of ABCC1 or ABCG2, while this uptake was increased to the level in the ABCB1-negative cells by a specific P-glycoprotein inhibitor, Tariquidar. We suggest that TP compounds can be used as highly sensitive, selective, non-toxic, and stable dyes to examine the functional expression and properties of the ABCB1 multidrug transporter, especially in microplate-based high-throughput flow cytometry assays. In addition, we demonstrate the applicability of the TP dyes to efficiently select and separate even a very low number of Pgp-expressing intact cells.

## 1. Introduction

Members of the ATP-binding cassette (ABC) transporter superfamily play important roles in the active transport of endo- and xenobiotics across the cell membranes and tissue barriers. The ABCB1 transporter (Pgp), has a major effect on the absorption, distribution, metabolism, excretion, and toxicity (ADME-Tox) of a large number of pharmacological agents, and causes cancer multidrug resistance by preventing the access of intracellular targets by various antineoplastic agents [1]. The other two major multidrug membrane transporter ABC proteins are the ABCC1 (multidrug resistance protein 1, MRP1) and the ABCG2 (breast cancer resistance protein, BCRP), and the selective functional diagnostics of these transporters is important both in drug development and clinical diagnostics [2,3,4,5,6,7].

ABCB1-Pgp was the first recognized human ABC multidrug plasma membrane transporter, [8,9] and this 170 kDa polypeptide is predominantly expressed in the intestine, blood-brain barrier, liver, pancreas, and kidney [4,6,8,10]. Pgp exports neutral or positively charged hydrophobic compounds from cells, thereby protecting them from drugs and xenobiotics [11]. Variable cellular expression of Pgp significantly affects the distribution and elimination of drug molecules and the response to chemotherapeutic agents, including newly developed, targeted anticancer drugs [12]. The role of Pgp has also been implicated in Alzheimer’s disease and chronic renal failure [13,14,15]. However, currently, there is no selective, non-toxic fluorescent reporter compound available for the functional studies of Pgp, especially if an automated analysis of multiple samples is required. Most reporter dyes used in ABC transporter diagnostics (e.g., Calcein-AM, Hoechst 33342, DCV or Phengreen SK-diacetate) are not selective for Pgp, and these dye assays require special conditions (e.g., stopping with ice-cold buffer, storage on ice until and during the measurement), making it difficult to automate the diagnostic procedures [2].

The TO-PRO family of dyes, identified as „cell membrane–impermeant cyanine nucleic acid stains” [16], belongs to the family of cyanine monomers (see Supplementary Figure S1), consisting of two positively charged and one intercalating unit. TO-PRO™-1 (TP1) has a one-carbon unit conjugating chain, while TO-PRO™-3 (TP3) has a three-carbon conjugating chain, and both compounds bind to various nucleic acids with high affinity. These compounds become highly fluorescent upon binding to nucleic acids, and have relatively narrow emission bandwidths, thus facilitating multicolor applications in imaging and flow cytometry. The nucleic acid bound fluorescence of these dyes is determined by the length of the conjugating chain—TP1 has a green fluorescence, TP3 shows a red fluorescence [17,18,19].

Although it has been claimed in the respective literature and catalogs that these dyes do not penetrate the membranes of living cells, a member of the family (YO-PRO-1) has been shown to cross the membranes of apoptotic cells or the P2XR7 receptor channels [20]. Still, the main applications of these dyes are live/dead cell separation or direct nucleic acid analysis. TP3 is exceptionally widely used as a viability dye, and to study viruses [21], bacteria [17], or to detect peripherals of the nervous system after fixation and permeabilization [22].

In the present work, we document that the TP dyes slowly penetrated the membranes of intact mammalian cells, and inside the cells they were trapped by binding to nucleic acids and become fluorescent. We focused here on TP1 and TP3, and show that these compounds were non-toxic to the cells and their fluorescence was stable for use in flow cytometry. Moreover, these dyes were actively extruded by the ABCB1/Pgp transporter, while not by the ABCC1 or ABCG2 multidrug pumps. Following TP accumulation by measuring cellular fluorescence and the concomitant use of PgP inhibitors makes this assay a simple, versatile, and sensitive tool for a selective functional evaluation of the ABCB1/Pgp transporter in human cells. Potential applications for efficient sorting of Pgp expressing cells, and the use of this method in high-throughput studies, are also presented.

## 2. Results

In this study, we examined the potential interactions of ABC multidrug transporters with TO-PRO™-1 and TO-PRO™-3 nucleic acid staining fluorescent dyes. These compounds are considered as membrane-impermeable, when used in short-term (maximum few hours) incubations. When the cell membrane becomes permeable in the dead cells, both of these dyes rapidly accumulate in the cells and become fluorescent upon binding to nucleic acids. Therefore, these compounds are widely used as viability markers in flow cytometry or for visualizing the cell nuclei after fixation and permeabilization of the cells.

### TP1 or TP3 Accumulation in Live Cells—Effects of ABC Transporters

First, we measured the long-term (more than 4 h and up to 24 h) accumulation and fluorescence of TP1 and TP3 in various live cell types, by using microplate-based flow cytometry. Then, we examined the effects of the presence of the three key multidrug transporters, ABCG2, ABCB1, and ABCC1 in the cells on dye accumulation. In these assays, we co-stained the cells with a short propidium iodide (PI) exposure to exclude dead cells.

**Figure 1:**
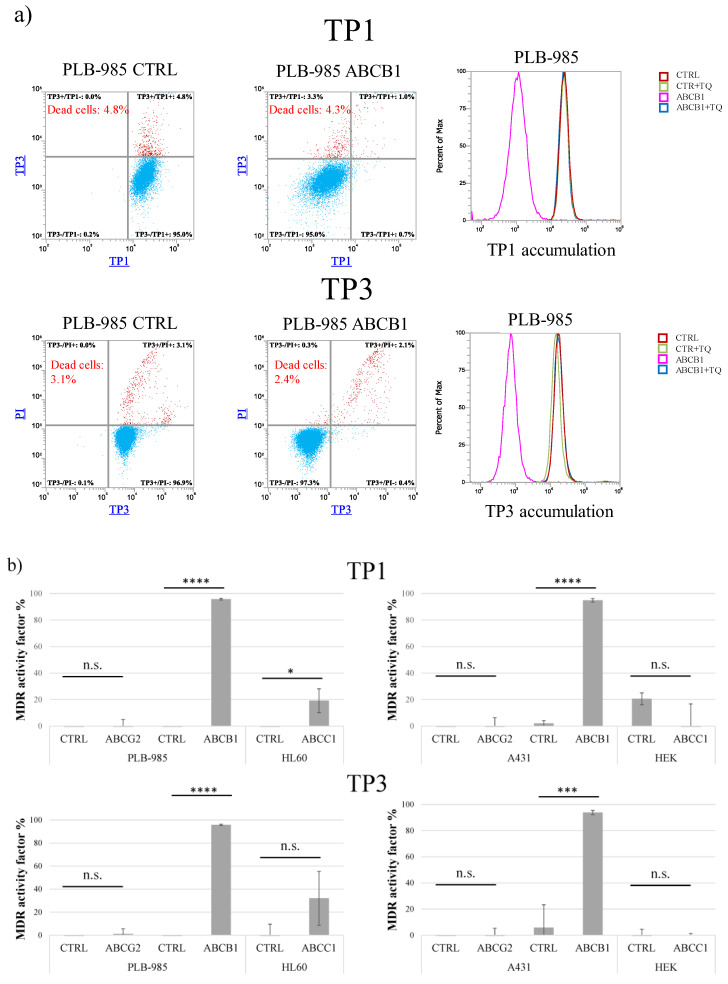
Uptake of TP dyes in human cells—effects of ABCG2, ABCB1, and ABCC1 expression in various cell types, measured by flow cytometry. (**a**) Uptake of 500 nM TP1 or 100 nM TP3 in PLB-985 control and ABCB1-expressing cells was measured after incubation for 24 h, at 37 °C. TP3 fluorescence was measured by Attune NxT flow cytometer with red laser (638 nm) in the RL1 channel (emission filter: 670/14 nm). Dead cells were identified based on PI staining (488 nm blue laser, emission filter: 695/40 nm). TP1 signal was measured by Attune NxT flow cytometer with blue laser (488 nm) in the BL1 channel (emission filter: 530/30 nm). Dead cells were identified based on TP3 staining (added immediately before measurement). Dead cells are labeled red, live cells are labeled blue. Experiments were repeated at least three times, the result of one representative experiment is shown. (**b**) MDR activity factors (see Methods) calculated by measuring inhibitor-sensitive TP1 or TP3 accumulation in various human cells, expressing ABCG2, ABCB,1 or ABCC1. Upper row: TP1 measurements, lower row: TP3 measurements. The specific inhibitors used were 2.5 μM KO143, ABCB1 by 250 nM TQ, and ABCC1 by 10 μM IM. SD +/- values are indicated. Statistical analysis was performed by Student’s t-test. n.s. not significant * *p* < 0.05 *** *p* < 0.001 **** *p* < 0.0001 (*n* = 3).

As shown in Figure 1, after a relatively long-term incubation (24 h at 37 °C), both TP1 and TP3 significantly penetrated into the living cells which do not express ABCB1/Pgp. This accumulation occurs even in cells which express ABCC1 or ABCG2, while the presence of ABCB1 practically eliminates this accumulation.

As documented in Panel a, cellular fluorescence after TP1 and/or TP3 incubation was well measurable in the PLB-985 control cells, while there was very little accumulation in the PLB-985-MDR1 cells. As indicated by the histogram graphs in Panel a, this latter accumulation was greatly increased, up to the level seen in the control cells, upon the addition of the ABCB1/Pgp inhibitor, tariquidar.

Figure 1b demonstrates the quantitation of the inhibition of TP1 (upper row) or TP3 (lower row) accumulations, respectively, in cell types expressing various ABC multidrug transporters. The calculated MDR activity factors show the relative efficiency of inhibiting dye accumulation by the respective ABC transporters, as compared to those seen by the addition of their selective inhibitors (see Methods and refs [23,24]). These measurements show that only ABCB1/Pgp expressing cell lines were capable of extruding either TP1 or TP3, thus reducing cellular fluorescence in an inhibitor-sensitive manner. The slight TP1/TP3 transport activity seen in the HL60-ABCC1 cells, and not in other ABCC1-expressing cells, may have been caused by a low level of ABCB1 in this drug-selected cell line.

In order to establish optimum conditions for assessing cellular TP uptake and ABCB1 function, we analyzed the time-dependence of the TP uptake in various cell types, (see Supplementary Figure S2.). As shown, when TP uptake was measured in the PLB-985 cells (in the presence of 200 nM or 500 nM TP1 in the culture media), the increase in cellular TP fluorescence at 37 °C was saturated after about 16 h. A similar saturation of cellular fluorescence was observed when 25 nM or 100 nM TP3 was applied. Similar time-dependent saturation curves were obtained in PLB-985 cell lines (Supplementary Figure S2).

**Figure 2:**
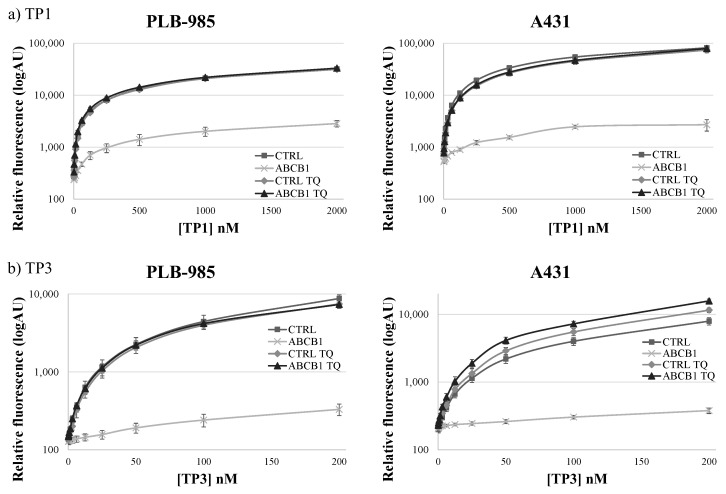
Concentration dependence of TP1 (**a**) or TP3 (**b**) dye uptake in PLB-985 and A431 control cells and in those overexpressing ABCB1. The CTRL (■) and ABCB1-expressing (×) cells were incubated with increasing concentrations of the dyes at 37 °C, for 24 h. The rhombuses (◊: CTRL) and the triangles (▲: ABCB1) demonstrate TP1 or TP3 accumulation in the presence of 250 nM TQ, a specific ABCB1 inhibitor. Dead cells were identified on the basis of rapid TP3 dye (for TP1) or PI staining (for TP3). +/- SD values are indicated (*n* = 3).

As shown in Figure 2, in the PLB-985 and A431 cells at low (200–500 nM of TP1 or 50–200 nM of TP3) concentrations, the presence of the ABCB1 protein in the cell membrane caused a major difference in the amount of the accumulated TP fluorescence. At increasing TP concentrations, the difference in cellular fluorescence caused by the ABCB1 protein still increased, while the ratio of the fluorescence in the absence and presence of ABCB1, respectively, did not increase. Based on these experiments, the suggested best conditions for assessing ABCB1/Pgp activity, in 24 h incubation periods at 37 °C, are 500 nM for TP1 and 100 nM for TP3.

In contrast to previously applied dye accumulation assays, due to the slow accumulation and low toxicity of TP1 or TP3, the differences in the long-term accumulation could be properly measured in a microplate-based system. Moreover, in a wide concentration range, TP1 and TP3 accumulation within 24 h showed no toxic effects regarding cell viability or cell growth (see Supplementary Figure S3). Therefore, in our further experiments we used a TP1 concentration of 500 nM, or a TP3 concentration of 100 nM, and 24-h incubation at 37 °C in 96-well microplates, providing optimum conditions for the functional ABCB1/Pgp assays.

In the following experiments, we examined the potential applicability of the fluorescence-based TP1 or TP3 accumulation assays for the sorting and selection of low number of cells expressing the ABCB1 protein. We compared the TP-based assays in this regard to the established DCV and the Calcein AM assays, previously used for similar purposes.

**Figure 3:**
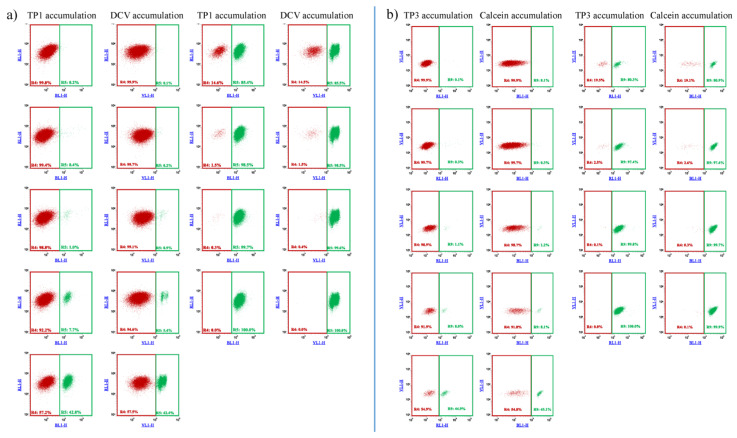
Flow cytometry detection of TP1 (**a**) or TP3 (**b**) accumulation in human PLB-985 cells: recognition and separation of control PLB-985 cells and PLB-985 cells expressing the ABCB1 transporter, respectively. Comparison to cell recognition by DCV accumulation. Control PLB-985 cells (green) and PLB-985 cells expressing wild-type ABCB1 (red) were mixed in various ratios, from 0.2% to 99.8%, respectively. TP1 accumulation was measured after incubation with 500 nM TP1 for 24 h at 37 °C. DCV accumulation was measured after the addition of 1 µM DCV (incubation for 1 h, at 37 °C). TP3 accumulation was measured after incubation with 200 nM TP1 for 24 h at 37 °C. Calcein accumulation was measured after the addition of 250 nM Calcein AM (incubation for 15 min at 37 °C).

As documented in Figure 3, and in Supplementary Figure S4, on the basis of the difference in TP1 or TP3 accumulation, ABCB1-expressing cell populations, representing even less than 1% of the total cell mixture, could be visualized and separated by flow cytometry. These experiments show that the relatively simple and optimized 96-well-based TP assays were similarly efficient for the recognition and separation of ABCB1/Pgp expressing cells as the previously applied less convenient and less selective fluorescence-based assays.

**Figure 4:**
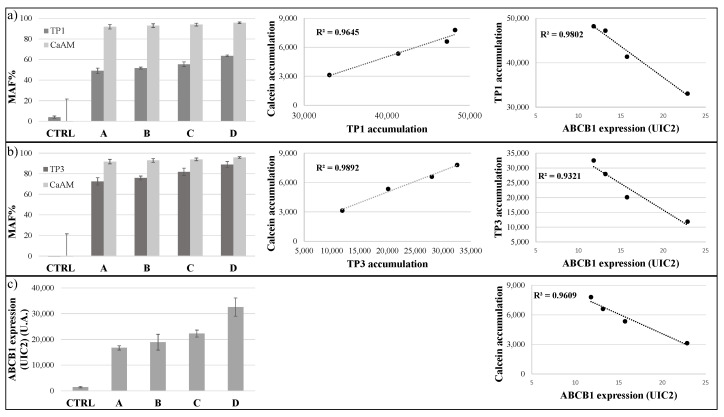
TP sensitivity assay in the MES-SA cell line. Uptake of 500 nM TP1 ((**a**), dark gray) or 100 nM TP3 ((**b**), dark gray) in the control and different ABCB1-expressing MES-SA cells was measured after 24 h incubation at 37 °C. Calcein accumulation ((**a**,**b**), light gray) was measured in control and ABCB1 expressing MES-SA cells after the addition of 250 nM Calcein-AM. MAF values were calculated as described in Methods. In all cases, Tariquidar (TQ, 250 nM) was applied as the selective inhibitor of ABCB1. Determination of cell surface expression of ABCB1 was performed by UIC2 antibody labeling (**c**). ABCB1 expressions were estimated relative to that in the CTRL cells. A scatter plot shows the relationship between the accumulation of TP1 or TP3 and the accumulation of Calcein, or the membrane expression levels of ABCB1. The scatter plots and the corresponding regression lines and linear regression coefficients (R^2^) were calculated by Excel (+/- SD values are indicated, *n* = 3).

In our following experiments, we examined how the TP-based functional assays correlate with the variable expression levels of the ABCB1/Pgp. In order to explore this question, we used MES-SA cells with no or increasing levels of ABCB1 expression. As shown in Figure 4, control and ABCB1-expressing MES-SA cells were incubated with 500 nM TP1 or 100 nM TP3 for 24 h at 37 °C, while in other experiments with 250 nM CaAM, for 15 min at 37 °C. The MDR activity (MAF) values were calculated by the inhibitor-sensitive increase in cellular fluorescence in the case of TPs and Calcein AM.

Interestingly, the MAF values obtained by measuring Calcein accumulation were less sensitive to the actual expression levels, probably because even low ABCB1 expression levels caused a maximum inhibition of Calcein AM uptake. The best correlation with the low levels of ABCB1 in the MES-SA cell membranes, measured by specific monoclonal antibody binding, was shown by the TP1 uptake (R^2^ = 0.9802). TP3 accumulation was somewhat less sensitive to the low levels of ABCB1 expression, giving high MAF values even at low membrane ABCB1 levels (R^2^ = 0.9321). Calcein accumulation was similar to TP3 accumulation (R^2^ = 0.9609). The relationship between the accumulation of the cyanine dyes and the accumulation of Calcein was strong, although TP1 accumulation was more sensitive, shown by the respective MAF values (TP3/Calcein R^2^ = 0.9892, TP1/Calcein R^2^ = 0.9645).

In addition to the flow cytometry studies, we also examined TP1 and TP3 accumulation in control A431 cells and in A431 cells expressing ABCB1, by using fluorescence-based confocal microscopy. In these experiments, to label the nuclei of the cells, we also included the staining of live A431 cells with DAPI.

**Figure 5:**
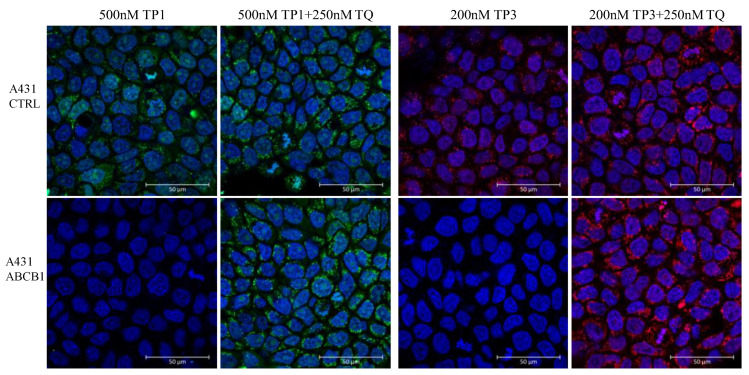
Fluorescent TP1 or TP3 accumulation in human A431 cells, examined by confocal microscopy. Effects of ABCB1 protein expression and the specific inhibition of the transporter function by Tariquidar. Cellular fluorescence was followed by confocal microscopy. TP1 fluorescence (green) or TP3 fluorescence (red) was examined after 24 h of the addition of 500 nM TP1 or 200 nM TP3 to the medium, either in the absence or presence of the transporter inhibitor (250 nM Tariquidar for ABCB1). The nuclei were counterstained with DAPI.

As shown in Figure 5, in a representative confocal microscopy study, the control A431 cells showed an intensive green or red signal due to dye accumulation in cell organelles, while the A431-ABCB1 cells practically did not accumulate TP1 or TP3. Upon the addition of the specific ABCB1 inhibitor, Tariquidar, cellular fluorescence in the ABCB1 expressing cells was greatly increased, while there was no significant change in the fluorescence level in the control cells. Interestingly, TP1 or TP3 in the live cell preparations did not show a concentrated nuclear staining (as expected based on studies in permeabilized cells), while fluorescence was observed in many cellular organelles containing nucleic acids (see Supplementary Figure S5).

## 3. Discussion

ABCB1/Pgp is an ATP-dependent plasma membrane efflux pump which plays a crucial physiological role in protecting tissues from toxic xenobiotics and endogenous metabolites. ABCB1/Pgp is also an important factor in cancer multidrug resistance, potentially protecting the transporter-expressing tumor cells from targeted chemotherapeutic agents. Several ABCB1 substrates have already been identified, and these are structurally unrelated hydrophobic and/or amphipathic compounds, including anticancer agents, peptides, or lipid-like compounds.

Some of the transported substrate compounds of ABC multidrug transporters are fluorescent and these are widely used for functional assays. Intracellular fluorescence of some compounds, when the dyes become fluorescent only after cellular cleavage (e.g., Calcein-AM or PhenGreen SK diacetate) and are trapped by a low efflux rate of the free dye, are certainly useful for multidrug transporter activity detection (see [2,24]). Several nucleic acid staining dyes, including Hoechst33342 or DCV, become highly fluorescent only after binding to intracellular DNA or RNA; thus, this intracellular trapping can also be efficiently used for monitoring an active dye extrusion by the transporters [4]. However, these reporter dyes are mostly non-selective and, because of the relatively short-term responses, are not suitable for high-throughput, microplate-based flow cytometry measurements. In this work our aim was to identify novel, specific ABCB1 substrate dyes, which also meet this latter requirement.

TP1 and TP3 are widely applied as viability dyes, as in short time incubations they do not permeate into intact cells and can be used for recognizing dead cells with leaky membranes, or fixed and permeabilized cells, through their binding to nucleic acids. In this study, we showed that these compounds slowly penetrated and accumulated in intact human cells, while the membrane expression of the ABCB1/Pgp transporter greatly reduced this accumulation. Importantly, the addition of specific ABCB1/Pgp inhibitors prevented this dye extrusion and the expression of the ABCG2 or the ABCC1 multidrug transporter did not reduce cellular TP1 or TP3 accumulation. We document that dye penetration and transporter extrusion were time- and concentration-dependent, and the accumulation of TP1 or TP3 was not toxic to the cells (see Supplementary Figure S3). Moreover, TP1 or TP3 extrusion by ABCB1/Pgp correlated with the membrane expression level or by the activity of the ABCB1 transporter, measured by monoclonal antibody staining and/or the Calcein assay. We also show that by using TP accumulation, even very low numbers of ABCB1 transporter-positive live cells could be distinguished and sorted out from mixed cell populations, and the assay was applicable for high-throughput studies in a stable microplate-based system.

Based on our studies, we suggest that the key application of the TP accumulation assay in intact cells should be based on flow cytometry and not by direct cellular imaging methods. When we examined the intracellular binding of TP1 or TP3 in the live cell preparations by fluorescence microscopy, this method did not show a high level of nuclear staining (as expected, based on studies in permeabilized cells), and fluorescence was observed in various cellular organelles, containing nucleic acids. Thus, intracellular membranes in live cells may have variable permeabilities for the TP dyes, complicating the quantitative evaluation of cellular dye accumulation. We note here that in our experiments vesicular TP transport studies by ABC-transporter expressing membrane vesicle could not be efficiently performed, as TP fluorescence would require nucleic acid binding and trapping in the membrane vesicles. TP dyes also produced only a small effect in the membrane ATPase assays.

As a summary, here, we document an application of TP1 or TP3, previously applied as “viability dyes”, for an efficient functional detection of the ABCB1/Pgp transporter in intact live cells by flow cytometry. The TP1 or TP3 uptake assays, complemented with the use of selective transporter inhibitors, provide new, highly sensitive, extremely stable, microplate-based, potentially high-throughput tools to examine the functional properties of the ABCB1 multidrug transporter and to efficiently select and sort transporter expressing cells.

## 4. Materials and Methods

### 4.1. Materials

TO-PRO™-1 Iodide and TO-PRO™-3 Iodide were purchased from Thermo Fischer Scientific (Waltham, MA, USA). KO143 was obtained from Tocris Bioscience (Bristol, UK). Indomethacin (IM) was purchased from Sigma-Aldrich-Merck (St. Louis, MO, USA). Tariquidar (TQ) was a kind gift from Dr. S. Bates (NCI, NIH). Calcein AM (Ca-AM) and Vybrant™ DyeCycle™ Violet Stain (DCV) were bought from Thermo Fischer Scientific (Waltham, MA, USA). The DAPI, Mitotracker Green and Lysotracker Green used to make microscopic images are from Thermo Fischer Scientific (Waltham, MA, USA). Components of phosphate buffered saline were obtained VWR (Radnor, PA, USA). All other materials, if unless otherwise were purchased from Thermo Fischer Scientific (Waltham, MA, USA).

### 4.2. Cell Lines

PLB-985 myelomonocytic and A431 skin derived epidermoid carcinoma cell lines, stably expressing the ABCG2 or the ABCB1 protein were generated by using a retroviral transduction system [2,25,26,27]. HEK-293 human embryonic kidney and HL-60 human promyelocytic leukemia cell lines stably expressing ABCC1 were also generated by retroviral transduction (HEK-293-ABCC1) or drug selection (HL60-ABCC1) [28]. We have also examined MES-SA (human sarcoma) cells, a gift from G. Szakács, stably expressing the ABCB1-Pgp protein at variable levels [29]. Expression of ABCB1, ABCC1 and ABCG2 was examined by immunostaining and subsequent flow cytometry analysis, using UIC2, QCRL3 and 5D3 antibodies, respectively (see Supplemental Materials).

### 4.3. Flow Cytometry

The transport activity of the ABC transporters was measured by Attune NxT flow cytometer (Thermo Fischer Scientific, Waltham, MA, USA) equipped with a violet (405 nm,) a blue (488 nm) and red (638 nm) lasers. The Calcein and TO-PRO-1 signal was detected in the BL1 channel (emission filter: 530/30 nm), TO-PRO-3 signal was detected in the RL1 channel (emission filter: 670/14 nm), the DCV signal was detected in VL1 channel (emission filter: 440/50). The live/dead marker was propidium-iodide (PI), which was detected in the BL3 channel (emission filter: 695/40 nm). The PE direct labeled UIC2 signal was measured in the BL2 channel (emission filter: 590/40 nm).

### 4.4. Microplate-Based TO-PRO-1 or TO-PRO-3 Accumulation Measurements by Flow Cytometry

For the microplate-based TP1 or TP3 accumulation measurements control and ABCB1-expressing A431 cells were seeded (3 × 10^4^ cells in 100 µL final volume/well) onto 96-well plates and cultured for 24 h at 37 °C, 5% CO_2_. The treatment was next day. The control and ABCB1-expressing PLB-985 cells were seeded (3 × 10^4^ cells in 100 µL final volume/well) onto 96-well plates and treated. At the end of the 24-h treatment period (except for time dependence), we supplemented the cells with 100 µL of complete medium containing 5 μg/mL propidium iodide (PI). Finally, fluorescence was measured at room temperature using an Attune Nxt flow cytometer with plate reader. All experiments were performed at least three times.

For assessing transporter inhibition, the ABCG2 transporter function was inhibited by 2.5 μM KO143, ABCB1 by 250 nM TQ, and ABCC1 by 10 μM IM. The cells (3 × 10^4^) were incubated with 500 nM TP1 or 100 nM TP3 with or without inhibitors, 24 h at 37 °C. In order to follow for the microplate-based time-dependent accumulation of TP1 or TP3, control and ABCB1-expressing PLB-985 cells were incubated in the culture medium with various concentrations of TP1 or TP3, with or without transporter inhibitor, at 37 °C, for 15 min to 24 h. For assessing the TP1 or TP3 concentration dependence PLB-985 or A431 cells, expressing the ABCB1 transporter, were incubated in culture media with 0.2–2000 nM of TP1 or 0.2–200 nM of TP3 at 37 °C for 24 h. In order to follow the time-dependent accumulation of TP1 or TP3 the control and ABCB1-expressing PLB-985 were incubated with 500 nM TP1 or 100 nM TP3 with or without inhibitors, 0–24 h at 37 °C.

### 4.5. Calcein Assay

The cellular accumulation of the fluorescent free Calcein in control and ABCB1-expressing cell lines was measured by the Calcein assay. The cells were incubated with 250 nM Calcein AM with or without 250 nM Tariquidar (TQ), as the specific ABCB1 transporter inhibitor, in DPBS (1 g/L D-glucose with phosphate buffered saline) for 15 min at 37 °C. Dye uptake was stopped by the addition of 250 μL ice-cold DPBS, and the cells were kept on ice until the measurements.

### 4.6. DCV Assay

The transport activity of control and ABCB1-expressing cell lines was measured by the DCV assay [30]. The cells were incubated with 1 μM DCV with or without 250 nM TQ in DPBS for 1 h at 37 °C. Dye uptake was stopped by the addition of 100 μL ice-cold DPBS, the cells were kept on ice until the measurements.

### 4.7. Membrane Expression Studies

We determined the cell surface expression of the control and different ABCB1-expressing MES-SA cell lines, control and ABCB1-expressing PLB-985 and A431 cell lines by using an ABCB1-specific monoclonal antibody, UIC2. Antibody labeling was performed with the UIC2 mouse monoclonal antibody conjugated with Phycoerythrin (PE) (Sony Biotechnology, San Jose, CA, USA). The live/dead marker was propidium-iodide (PI). The measurements were carried out in an Attune Nxt flow cytometer after gating for live cells.

The cell surface expression of the control and ABCC1-expressing PLB-985 or A431 cell lines were determined by the ABCC1-specific QCRL3 mouse monoclonal antibody, conjugated with A488 (Sony Biotechnology, San Jose, CA, USA).

The cell surface expression of the control and ABCG2-expressing PLB-985 or A431 cell lines was determined by the ABCG2-specific 5D3 mouse monoclonal (gift of Bryan Sor-rentino, Division of Experimental Hematology, Department of Hematology/Oncology, St. Jude Children’s Research Hospital) by adding 2.5 μM Ko143 inhibitor to achieve optimal antibody binding. Alexa Fluor 647-labeled IgG2b (Thermo Fisher, Waltham, MA, USA, cat. A-21242) was used as a secondary antibody. The expression of ABCC1 or ABCG2 transporters was measured by FacsCanto II flow cytometer (BD Bioscience, San Jose, CA, USA) equipped with a blue (488 nm) and red (633 nm) lasers. The QCRL3-A488 signal was detected in the FITC channel (emission filters: 530/30 nm), 5D3-A647 was detected in the APC channel (emission filters: 660/20 nm) (see Supplementary Material).

### 4.8. Flow Cytometry Data Analysis

Data analysis was performed using the Attune Acoustic Focusing Cytometer v.3.2.1. Software (Applied Biosystems, Life Technologies, Carlsbad, CA, USA). Results were expressed as median ± standard deviation. The MDR activity factor % (MAF%—see refs [23,24]) was calculated as follows: MAF% = (((MFI_inh_ − MFI_0_)/MFI_inh_) × 100), wherein MFI_inh_ and MFI_0_ are the median fluorescence intensity (MFI) with (inh) or without (0) inhibitor.

### 4.9. Confocal Microscopy Images

For confocal microscopy, the control and ABCB1- expressing A431 cells (5 × 10^5^) were treated with 200 nM TP3 or 500 nM TP1 with or without 250 nM TQ. At the end of the 24-h incubation, the cells were treated with 4 µM DAPI, 200 nM Mitotracker Green or 1 µM Lysotracker Green. The images were acquired by a Zeiss LSCM 710 microscope using a 63 × NA = 1.4 Plan Apo objective. Images were captured and analyzed by Zen2 (Blue edition, v3.1.) Software (Carl Zeiss Microscopy GmbH, Göttingen Germany). 

## 5. Patents

This work is currently under patent consideration.

## Data Availability

The datasets generated and analyzed during the current study are available from the corresponding authors on reasonable request. The data is currently not public due to patent considerations.

## Supplementary Materials

The following supporting information can be downloaded at: https://www.mdpi.com/article/10.3390/ijms231810599/s1.
