# Supplementary material for: Selective Fluorescent Probes for High-Throughput Functional Diagnostics of the Human Multidrug Transporter P-Glycoprotein (ABCB1)
Source: Int J Mol Sci. 2022 Sep 13;23(18):10599. doi: 10.3390/ijms231810599 (PMC9503576; doi:10.3390/ijms231810599)
Supplement: Supplementary file 1 [file ijms-23-10599-s001.zip › ijms-1893730-supplementary.pdf]

Selective Fluorescent Probes for High-Throughput Functional Diagnostics of the Human Multidrug Transporter P-Glycoprotein (ABCB1)

Supporting information

A

Name	Catalog #	MW *	Abs † (nm)	ϵ_{max} ‡ (cm^{-1} M^{-1})	Em † (nm)	QY§	Excitation Light Source (nm)
PO-PRO™-1	P3581	579	435	50,100	455	0.39	He-Cd 442
BO-PRO™-1	B3583	595	462	58,100	481	0.16	He-Cd 442
YO-PRO®-1	Y3603	629	491	52,000	509	0.44	Ar 488
TO-PRO®-1	T3602	645	515	62,800	531	0.25	Ar 514
JO-PRO™-1	J11373	630	530	94,400	546	0.38	Nd: YAG 532
PO-PRO™-3	P3585	605	539	87,900	567	0.57	He-Ne 543
BO-PRO™-3	B3587	621	575	80,900	599	0.62	Kr 568
YO-PRO®-3	Y3607	655	612	100,100	631	0.16	He-Ne 594
TO-PRO®-3	T3605	671	642	102,000	661	0.11	He-Ne 633
TO-PRO®-5	T7596	697	748	108,500	768	ND	

* Molecular weight. † Absorption and fluorescence emission maxima. ‡ Molar extinction coefficient. § Fluorescence quantum yield determined relative to fluorescein in 0.1 M NaOH (QY = 0.92). Abs, Em, ,max and QY determined for DNA complexes in 10 mM Tris, 1 mM EDTA, 50 mM NaCl, pH 7.4. The spectral appearance of some dyes may be slightly altered inside cells. **ND** = Not determined.

B

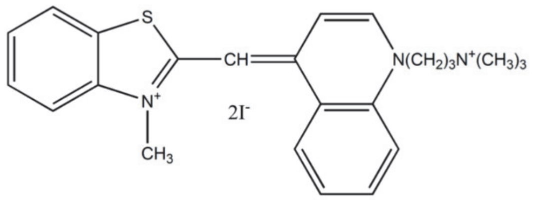

TO-PRO™-1

C

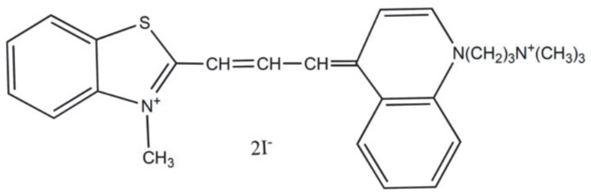

TO-PRO™-3

Figure S1. A, Fluorescence spectra of monomeric cyanine nucleic acid stains. B, and C, Chemical structure of TO-PRO-1 and TO-PRO-3, respectively.

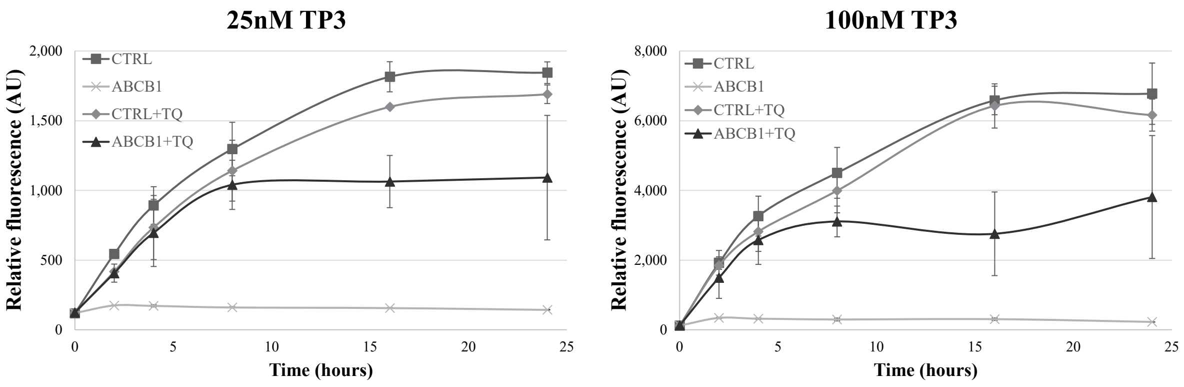

Figure S2. Time dependent accumulation of TP3 dye fluorescence in PLB-985 cells overexpressing ABCB1. The CTRL (■) and ABCB1 (×) cells were incubated with 25 nM or 100 nM TP3 from 0-24 h, at 37 °C. The rhombuses (◊:CTRL) and triangles (▲:ABCB1) demonstrate TP3 accumulation in the presence of 250 nM TQ, a specific ABCB1 inhibitor. Dead cells were identified based on PI staining. +/- SD values are indicated (*n*=3).

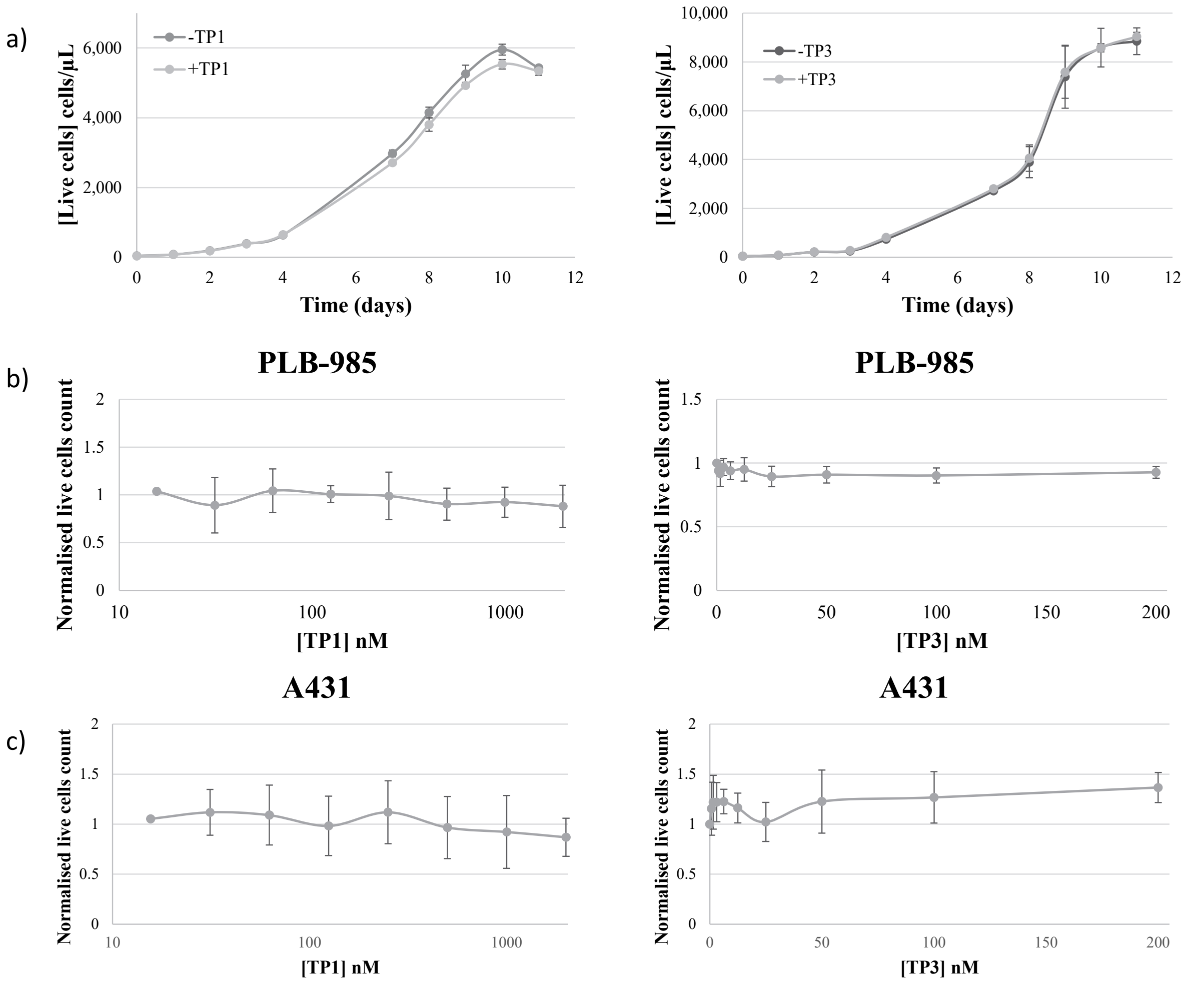

Figure S3. TP1 or TP3 toxicity assays. A, Effect of TP1 or TP3 accumulation on cell growth in PLB cells and PLB-ABCB1 cells. Cell growth was measured after 500 nM TP1 or 100 nM TP3 treatment for 24h at 37°C. B, C, Cytotoxic effects of TP1 or TP3 treatment in PLB-985 (B) and A431 (C) cells. The cells were pre-treated with the increasing concentrations of TP1 or TP3 for 24 h at 37 °C, then washed and cultured for 72 hours. Dead cells were identified based on TP3 or PI staining. +/- SD values are indicated (*n*=3).

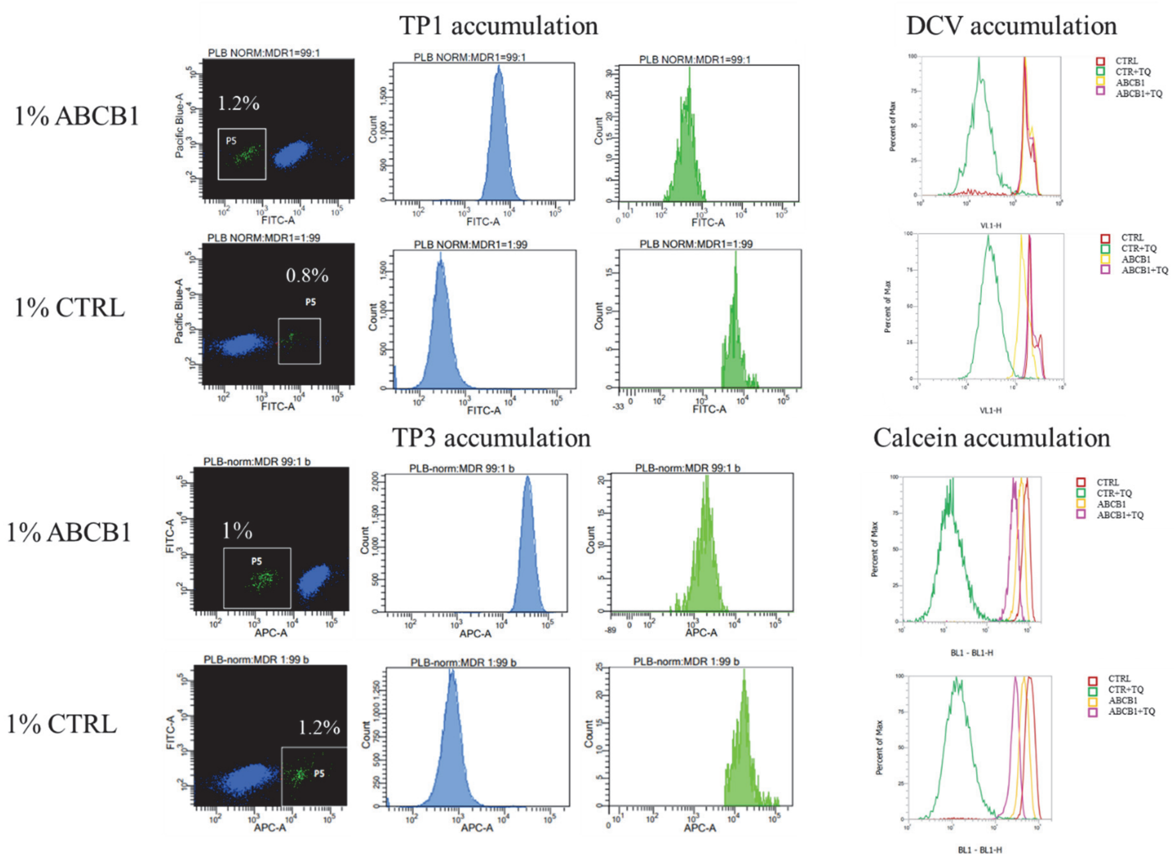

Figure S4. Flow cytometry detection of TP1 or TP3 accumulation in human PLB cells. Recognition and separation of control PLB cells and PLB cells expressing the ABCB1 transporter by cell sorting. Control PLB cells and PLB cells expressing ABCB1 were mixed in 99:1 or 1:99 ratios. Cells were incubated with 500 nM TP1 or 100 nM TP3 for 24h, 37°C. The mixed cell cultures were sorted by FACSAria III with blue laser (488nm) in the FITC channel (emission filter: 525/40 nm) and with red laser (633 nm) in the APC channel (emission filter: 660/20 nm). After cell sorting, the purity of the populations was examined by DCV accumulation (TP1) or Calcein accumulation (TP3).

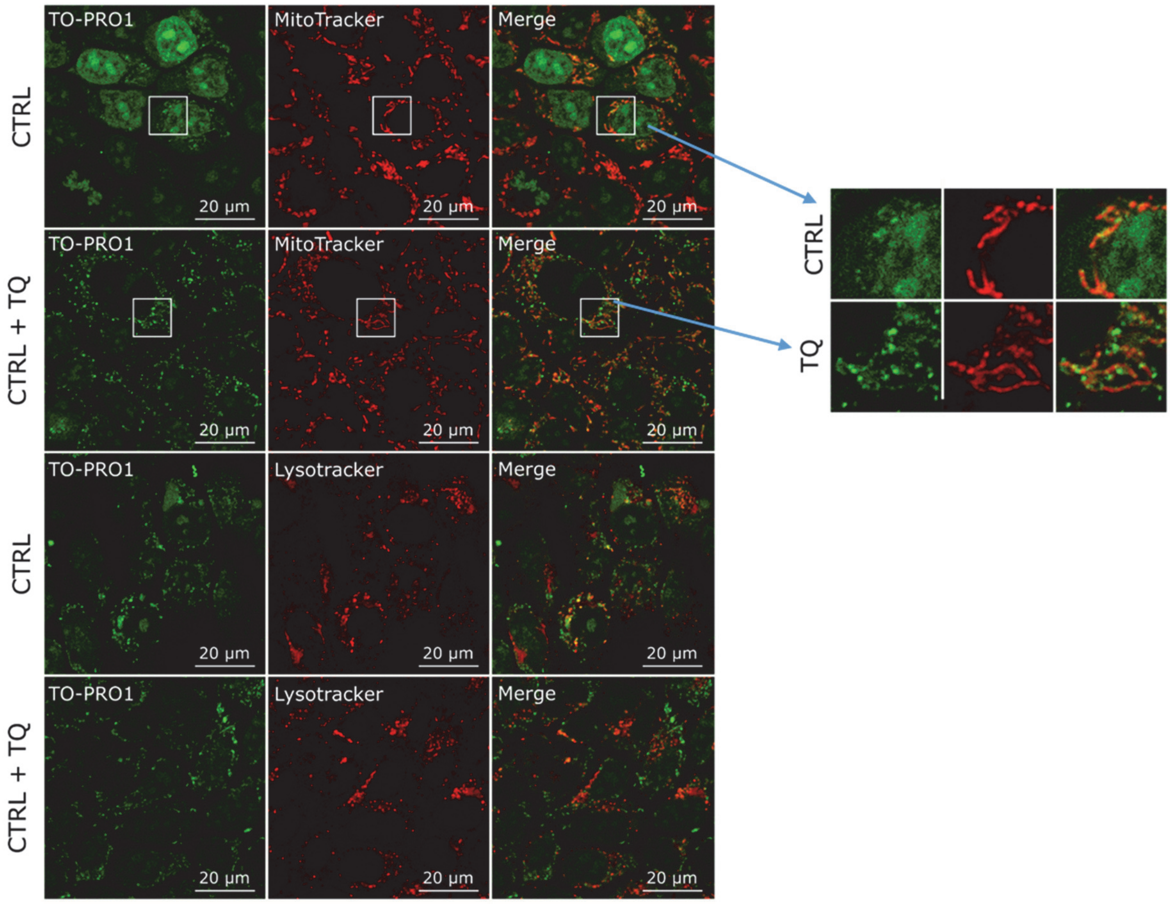

Figure S5. Co-localisation of TP1 staining with Mitotracker Red, a mitochondrion-specific marker in A431 CTRL cells. TP1 fluorescence (green) was examined after 24 h of the addition of 500 nM TP1 to the medium, either in the absence or presence of 0.25 µM tariquidar. After 24 h, the cells were co-stained with Mitotracker Red (red). Merging the red and green signals shows a yellow color in overlapping regions. The TP1 staining co-localised – especially after the addition of TQ - mostly (as punctate structures) with the mitochondria. The inset shows higher magnification of the specific area.

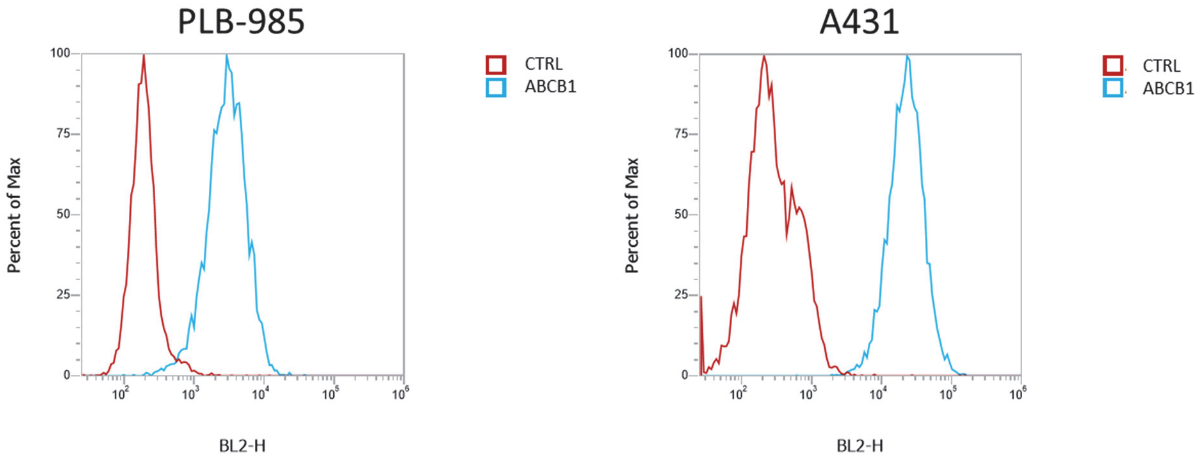

Figure S6. Flow cytometry analysis of cell surface membrane expression of ABCB1 in PLB-985 and A431 cells, respectively, detected by phycoerythrin labelled UIC2 antibody.

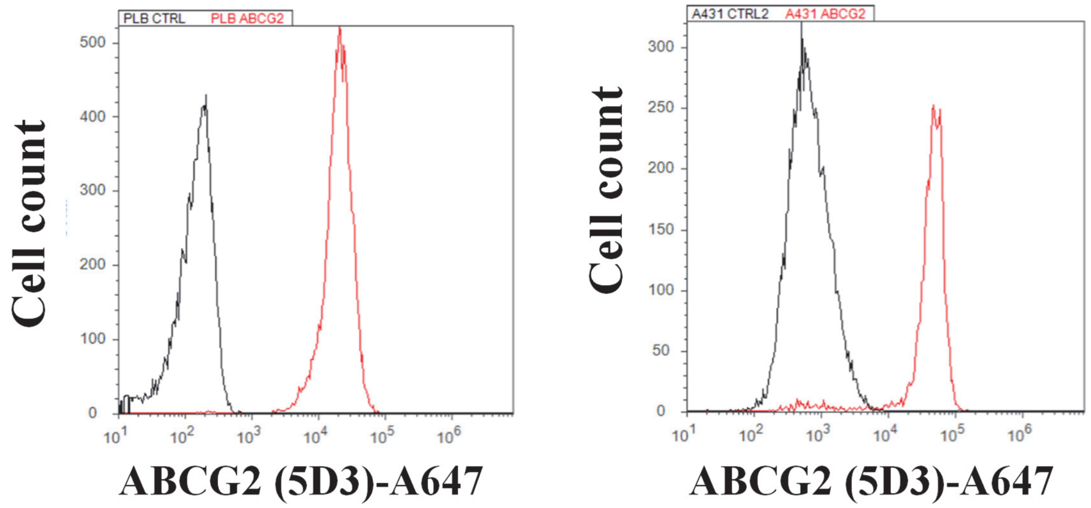

Figure S7. Flow cytometry analysis of cell surface membrane expression of ABCG2 in PLB-985 and A431 cells, respectively, detected by A647 labeled 5D3 antibody.

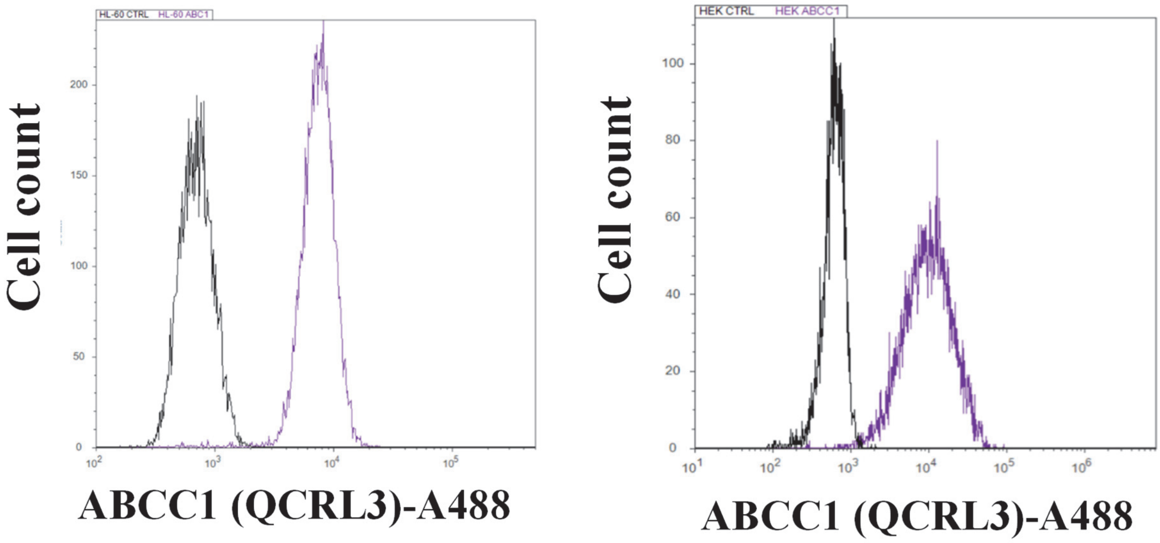

Figure S8. Flow cytometry analysis of cell surface membrane expression of ABCC1 in PLB-985 and A431 cells, respectively, detected by A488 labeled QCRL3 antibody.
